# Prognostic value of systolic short-term blood pressure variability in systolic heart failure

**DOI:** 10.1186/s40885-016-0051-z

**Published:** 2016-07-12

**Authors:** Matthieu Berry, Olivier Lairez, Joelle Fourcade, Jérôme Roncalli, Didier Carrié, Atul Pathak, Bernard Chamontin, Michel Galinier

**Affiliations:** Department of Cardiology, University Hospital of Rangueil, 1, Avenue Professeur Jean Poulhès, 31095 Toulouse, France; Cardiac Imaging Center, University Hospital of Toulouse, Toulouse, France; Rangueil Medical School, Toulouse, France; Department of Nuclear Medicine, University Hospital of Toulouse, Toulouse, France; Purpan Medical School, Toulouse, France; Department of Pharmacology, Purpan Medical School, Toulouse, France

**Keywords:** Heart failure, Blood pressure variability, Prognosis

## Abstract

**Background:**

Traditional cardiovascular risk factors in the general population are usually correlated to a better prognosis in patients with chronic heart failure (HF). Most of the studies show that blood pressure variability (BPV) has noxious effect on general population but data are missing for patients with systolic HF. The aim of this study was to assess the prognostic impact of short-term blood pressure variability (BPV) in systolic HF.

**Methods and results:**

We retrospectively studied 288 patients (60 ± 12 years-old; 79 % male) referred to our tertiary center of HF for the management of their systolic HF (left ventricular ejection fraction was 28 ± 9 %). All patients underwent ambulatory blood pressure monitoring (systolic BP: 110 ± 15; diastolic BP: 68 ± 10 and pulse pressure: 42 ± 11 mmHg) and the prognostic impact of BPV was collected with a mean follow-up of 4.4 ± 3.1 years. Twenty-five (9 %) patients were missing for follow-up. Among the others patients, 70 (27 %) cardiovascular events (cardiac deaths: 24 %; heart transplantation: 2 %) were recorded. By multivariate analysis BPV daytime (OR = 0.963, *p* = 0.033) and severe NYHA class (OR = 5.2, *p* < 0.0001) were found as independent predictors of cardiac event. Patients with a systolic daytime BPV under a cut-off value of 19 mmHg had the poorest prognosis with an OR for cumulative events of 1.65 (IC95 % 1.1–2.7; *p* < 0.04).

**Conclusion:**

BPV is simple tool and a predictor of cardiac events in patients with systolic HF.

## Background

If high blood pressure (BP), body mass index and cholesterolemia represent traditional cardiovascular risk factors in the general population, they are correlated to a better prognosis in patients with chronic heart failure (HF) [1–3]. In a meta-analysis, Raphael and al emphasized the paradoxical effect of higher systolic BP on mortality of patients with chronic HF, showing a decrease of 13 % in cardiovascular death for an increase of 10 mmHg in systolic BP [1]. For the last decades, the prognostic impact of each determinant of BP profile such as systolic BP, diastolic BP, pulse pressure (PP), BP variability (BPV) was essentially studied in patients with hypertension [4], but few in chronic HF. Thus, Rothwell et al. showed in a hypertensive population that the daytime BPV was a powerful predictor of stroke and coronary events [5]. The aim of the present study was to assess the prognostic impact of short-term BPV in chronic systolic HF.

## Method

### Population

Patients were retrospectively extracted from our local database of HF and including patients referred the exploration and the management of systolic HF in of the HF unit of the University Hospital of Toulouse from 1999 to 2006. Inclusion criterions were: age over 18 years old, one systolic HF event in life, systolic dysfunction defined by left ventricular ejection fraction <45 %, ambulatory monitoring of BP in at admission. Exclusion criterions were: patients with low flow or treated with intravenous drugs such as inotropic support, infection, dialysis and incomplete ambulatory monitoring of BP.

The study was approved by our local ethics committee.

### Twenty-four-hour ABPM

Twenty-four-hour ambulatory BP monitoring (ABPM) was performed as previous described in chronic HF [6], using the oscillometric method (Spacelabs 90207 device® [7]). Successive BP measures were performed every 15 min during daytime (7 am. to 9:59 pm.) and every 30 min during nighttime (10 pm. to 6:59 am.). BP measures were expressed in millimetres of mercury (mmHg). All devices for ABPM were placed on the right arms by a trained nurse 24-h after admission. Patients were instructed to relax the cuffed arm during the measure and received a diary to record unexpected events. The analysis of ABPM records were performed using Spacelabs software allowing us to extract systolic BP, diastolic BP, PP, BPV and Dip ratio daytime and nighttime BPV was calculated using the average difference between maxima and minima from each systolic BP measure to another. Nighttime BP dipping can be quantified as the ratio of mean nighttime (sleep) BP to mean daytime (awake) BP. The calculation formula was: BPV = (maximum systolic BP – minimum systolic BP)/2.

### Follow-up

Follow up was conducted using physician, patient or family phone contacts. Patients without news within the last month after the AMBP were considered as missing for follow-up. The composite endpoint was defined by the occurrence of cardiovascular events: cardiac death or heart transplantation.

### Statistical analysis

Continuous variables with a normal distribution are expressed as mean ± standard deviation (SD). To compare numerical data between two groups, paired and unpaired Student *t*-test was used when appropriate. Nominal variables were compared using either the *χ*^2^ or Fischer tests when appropriate. Univariate analysis of the predictive factors of cardiovascular event was performed using respectively log rank and cox methods for qualitative and quantitative variables. Kaplan Meier curve and log mantel cox method were used to illustrate prognosis of high and low level of systolic BP and VBP. Variables from the univariate analysis with *P* < 0.1 were included in the multivariable stepwise analysis to identify independent predictors of events. Two-tailed P-values <0.05 were considered statistically significant.

## Results

### Baseline characteristics

Two hundred and eighty eight patients (mean age 60 ± 12, 79 % male) with systolic HF were included in the study. The baseline characteristics are summarized in Table 1. Mean left ventricular ejection fraction was 28 ± 9 %. One hundred and thirty one (45 %) patients had ischemic cardiomyopathy, 123 (43 %) patients had primitive dilated cardiomyopathy, 37 (13 %) patients suffered from an alcohol-abused cardiomyopathy and 13 (5 %) patients had valvular heart diseases. There were 16 (6 %), 137 (47 %), 122 (42 %) and 13 (5 %) patients with NYHA stage I, II, III and IV, respectively.

**Table 1:** Baselines characteristics

N	288
Age, years	60 ± 12
Female, n (%)	61 (21)
BMI, Kg/m^2^	26 ± 4
NYHA class, n (%)	
I	16 (6)
II	137 (47)
III	122 (42)
IV	13 (5)
Left Ventricular Ejection Fraction, %	28 ± 9
Hypertension, n (%)	125 (43)
Diabetes, n (%)	61 (21)
Ischemic cardiomyopathy, n (%)	131 (45)
Primitive cardiomyopathy, n (%)	123 (43)
Valvular cardiomyopathy, n (%)	13 (5)
Congestive heart failure, n (%)	123 (42)
ACE inhibitors, n (%)	239 (83)
Beta-Blockers, n (%)	179 (62)
Spironolactone, n (%)	141 (49)

The usable of BP records during the 24-h monitoring was 87 ± 11 %. Regarding BP profile, mean systolic BP was 110 ± 15 mmHg, mean diastolic BP was 68 ± 10 mmHg and PP was 42 ± 11 mmHg during the 24-h monitoring. Mean BPV was higher on daytime versus nighttime (23 ± 9 vs. 18 ± 7 mmHg, *p* < 0.0001, respectively). Details of ABPM are shown in Table 2.

**Table 2:** ABPM among the overall population’s study

ABPM	24 h	Daytime	Nighttime
SBP, mmHg	110 ± 15	112 ± 16	107 ± 16
DBP, mmHg	68 ± 10	70 ± 10	65 ± 10
PP, mmHg	42 ± 11	42 ± 11	42 ± 11
BPV, mmHg	21 ± 7	23 ± 9	18 ± 7
Dip, mmHg	-	-	-6,6 ± 8

*BPV* blood pressure variability, *DBP* diastolic blood pressure, *PP* pulse pressure

### Follow-up and events

Twenty-five (9 %) patients were missing for follow-up.

Among the 263 others patients, 70 (27 %) cardiovascular events were recorded including 64 (24 %) cardiac deaths and 6 (2 %) heart transplantations with a mean follow up of 4.4 ± 3.1 years. The event group display a worse functional state (74 % vs. 38 %, *p* < 0.0001 for NYHA III/IV) with more often decompensate HF at admission (55 % vs. 38 %, *p* = 0.016) than the non-event group. This increase of congestive signs in the event group led to an increased of diuretics administration in comparison with to the non-event group (95 vs. 83 %, *p* = 0.013). However, there was no significant difference for left ventricular ejection fraction between the two groups (*p* = 0.19).

There was no difference for BP profile between patients with event and patients without event, except for daytime systolic BP (108 ± 16 vs. 113 ± 15 mmHg, *p* = 0.03, respectively), daytime diastolic BP (68 ± 9 vs. 71 ± 10 mmHg, *p* < 0.05, respectively) and daytime BPV (22 ± 6 vs. 24 ± 9 mmHg, *p* < 0.05, respectively). Results according to the occurrence of events are shown in Table 3.

**Table 3:** Events (cardiac death or heart transplantation) according to baseline characteristics (A) and blood pressure profile (B)

A.			
Baseline characteristics	Event (*n* = 70)	No event (*n* = 218)	*p*
Age, years	62 ± 12	59 ± 12	0.1
Male, n (%)	60 (86)	167 (77)	0.1
BMI	25.2 ± 3.6	26.2 ± 5.8	0.03
NYHA I &II, n (%)	18 (26)	135 (62)	<0.001
NYHA III&IV, n (%)	52 (74)	83 (38)	
LVEF, %	27 ± 10	29 ± 9	0.2
Congestive HF, n (%)	39 (55)	83 (38)	<0.05
Diabetes, n (%)	19 (27)	42 (19)	0.16
Hypertension, n (%)	22 (31)	103 (47)	0.02
Ischemic, n (%)	34 (49)	97 (44)	0.5
Cockcroft, ml/min	64 ± 24	72 ± 28	<0.05
Heart rate, bpm	79 ± 15	79 ± 18	0.8
ACE inhibitor, n (%)	54 (77)	187 (86)	0.09
ARA2, n (%)	7 (10)	12 (6)	0.19
Spironolactone, n (%)	34 (49)	106 (49)	0.99
B-blockers, n (%)	38 (54)	141 (65)	0.12
Ca-channel blockers, n (%)	1 (1)	12 (6)	0.15
Nitride, n (%)	12 (17)	43 (20)	0.6
Diuretics, n (%)	66 (94)	182 (83)	0.02
B.			
Blood pressure profile	Event (*n* = 70)	No event (*n* = 218)	*p*
SBP 24-h, mmHg	107 ± 15	111 ± 15	0.05
SBP daytime, mmHg	108 ± 16	113 ± 15	0.03
SBP nighttime, mmHg	104 ± 15	108 ± 17	0.13
DBP 24-h, mmHg	66 ± 9	69 ± 9	0.06
DBP daytime, mmHg	68 ± 9	71 ± 10	<0.05
DBP nighttime, mmHg	64 ± 10	66 ± 11	0.24
DIP, mmHg	3.4 ± 5	4.5 ± 7	0.23
PP daytime, mmHg	40 ± 11	42 ± 10	0.13
PP nighttime, mmHg	40 ± 11	42 ± 11	0.22
BPV daytime, mmHg	22 ± 6	24 ± 9	<0.05
BPV nighttime, mmHg	17 ± 6	18 ± 8	0.4

*ACE* angiotensin converting enzyme, *ARA2* angiotensin 2 receptor antagonist, *BMI* body mass index, *LVEF* left ventricular ejection fraction, *NYHA* New York Heart Association, *BPV* blood pressure variability, *DBP* diastolic blood pressure, *PP* pulse pressure

### Univariate predictors of cardiac event

Results of univariate analysis are summarized in Table 4. By univariate analysis, discriminative-predictive variables were NYHA stage III/IV (OR = 5.1, IC95 %: 3–8, *p* < 0.0001), LVEF < 25 %(OR = 1.96, IC95 %: 1.1–3.6, *p* = 0.01), congestive CHF (OR = 2.15, IC95 %: 1.3–3.5, *p* < 0 .002), decrease of natremia (1 mM/L;OR = 0.93, IC95 %: 0.870–0.990, *p* = 0.03), increase of the clearance of creatininemia (1 mL/min; OR = 0.988, IC95 %: 0.979–0.997, *p* = 0.01).

**Table 4:** Univariate analysis for cardiac events (cardiac death or heart transplantation)

A.			
Baseline characteristics	OR	IC95 %	*p*
Age	1.016	0.997–1.036	0.12
Male	0.644	0.33–1.259	0.19
BMI	0.954	0.903–1.007	0.09
NYHA III -IV	5.1	3–8.8	<0.0001
LVEF <25 %	1.959	1.073–3.575	0.01
Congestive	2.15	1.34–3.45	<0.002
Diabetes	0.65	0.38–1.1	0.08
Hypertension	0.493	0.297–0.817	0.005
Ischemic cardiomyopathy	0.859	0.538–1.373	0.5
Cockcroft ml.min¯^1^	0.988	0.979–0.997	0.01
Heart rate	0.999	0.984–1.014	0.9
B.			
Blood pressure profile	OR	IC95 %	*p*
SBP 24 h	0.979	0.962–0.996	<0.02
SBP daytime	0.977	0.961–0.994	<0.01
SBP nighttime	0.984	0.969–1	<0.05
DBP 24 h	0.974	0.948–1	0.05
DBP daytime	0.982	0.946–0.998	<0.04
DBP nighttime	0.985	0.962–1.008	0.2
DIP Systolic	0.987	0.955–1.02	0.4
DIP Diastolic	0.987	0.961–1.015	0.4
PP daytime	0.975	0.951–1	0.052
PP nighttime	0.978	0.955–1.002	0.07
Systolic BPV daytime	0.958	0.924–0.993	0.02
Systolic BPV nighttime	0.975	0.938–1.013	0.19

*BMI* body mass index, *LVEF* left ventricular ejection fraction, *NYHA*, New York Heart Association, *BPV* blood pressure variability, *DBP* diastolic blood pressure, *PP* pulse pressure

**Fig. 1:**
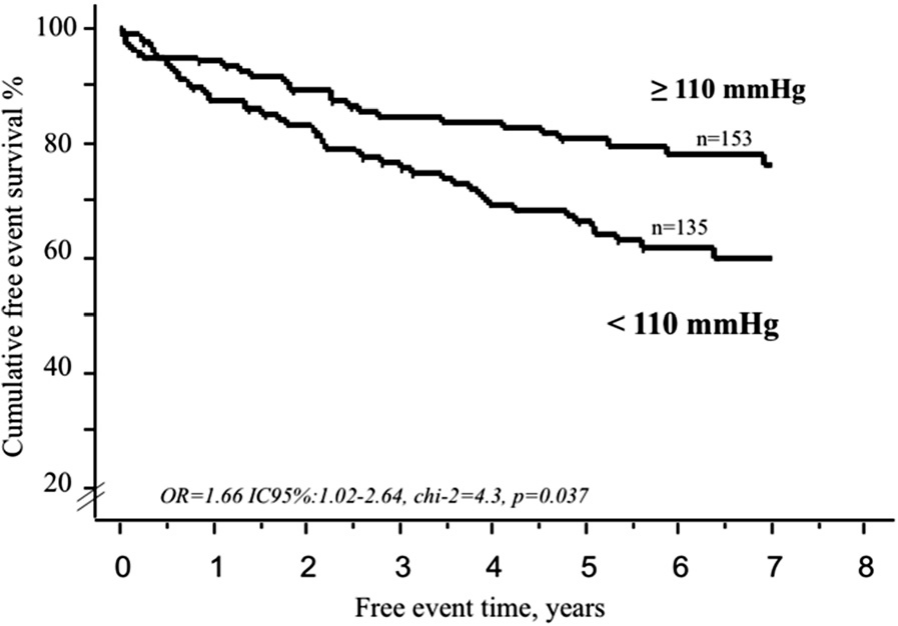
Mean daytime systolic blood pressure level and survival. This figure shows the impact of daytime systolic blood pressure level with a cut-off value of 110 mmHg on survival in patients with systolic heart failure

Regarding the BP profile, each 1 mmHg increase of 24-h systolic BP (OR = 0.979, IC95 %:0.962–0.996, *p* < 0.02), daytime systolic BP (OR = 0.977, IC95 %: 0.961–0.994, *p* < 0.01), nighttime systolic BP (OR = 0.984, IC95 %: 0.969–1, *p* < 0.05) daytime diastolic BP (OR = 0.982, IC95 %: 0946–0.998, *p* < 0.04) and systolic BPV (OR = 0.958, IC95 %: 0.924–0.993, *p* = 0.02) were correlated with a decrease of cardiac events. PP and systolic Dip had not significant impact in prognosis (Table 4B.) The Fig. 1 illustrates the predictive positive impact of a daytime SBP ≤ 110 mmHg on cumulative survival.

### Multivariate analysis

**Fig. 2:**
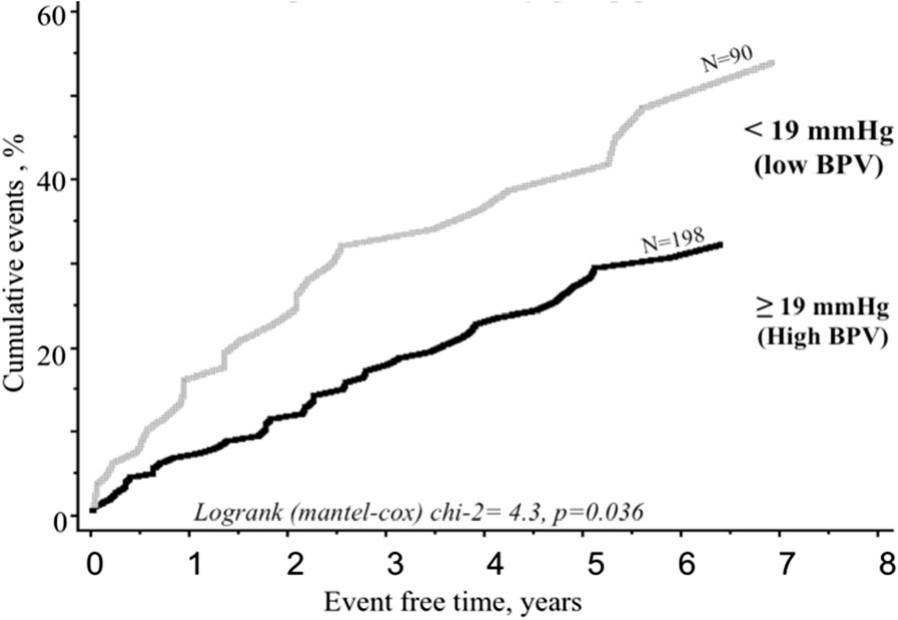
Blood pressure variability group patient. This figure shows the impact of blood pressure variability with a cut-off value of 19 mmHg on survival in patients with systolic heart failure

**Fig. 3:**
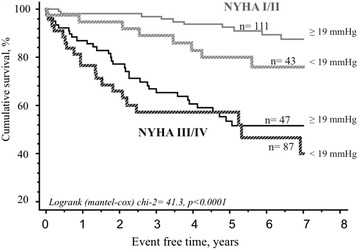
Impact of functional status and blood pressure variability on survival. This figure shows the relative impact of blood pressure variability according to NYHA stage on survival in patients with systolic heart failure

Daytime BPV, daytime SBP, nighttime SBP, daytime and nighttime PP for ABPM variables, and age, body mass index, diabetes and decompensate HF for clinical variables were included in stepwise multivariable cox model. Forward and backward stepwise allowed to characterize BPV daytime (OR = 0.963, *p* = 0.033) and severe NYHA class (OR = 5.2, *p* < 0.0001) as two independent predictors of cardiac events. As shown in Fig. 2, a systolic daytime BPV under a cut-off value of 19 mmHg had a poor prognostic impact with an OR for cumulative events of 1.65 (IC95 % 1.1–2.7; *p* < 0.04). The association of NYHA and BPV gave a better prognosis (Fig. 3).

## Discussion

Low daytime BPV (<19 mmHg) is found as an independent factor of cardiac death and/or cardiac transplantation. The daytime level of BPV, as systolic BP level, is correlated with the prognosis of patients with systolic HF. Previous study of Gibelin et al. [8] showed a worse prognosis of a low BPV in 154 patients with CHF. Our study confirms this result with a biggest recruitment during a mean follow up of 4.4 years. Thus, in comparison to previous studies on the prognostic value of BPV in hypertension, we have demonstrated a paradoxical prognostic impact of BP variability on cardiovascular event occurrence in systolic HF (cardiac death and heart transplantation).

### Determinant factors of BPV in CHF

Decrease of BPV in patients with CHF is well described. Radaelli et al. [9] demonstrated an alteration of BPV in nine patients with congestive CHF during exercise with a BPV of 15 and 41 mmHg^2^ in patients with CHF and healthy control subjects respectively [2]. The question that remained is: what mechanism(s) underlie(s) the decrease of BPV in CHF? If components involved in BP as cardiac output or systemic vascular resistance are well identified in patients with CHF, determinant factors of its variability are unclear. Intrinsic factors such as baroreflex sensibility [10], autonomic nervous system integrity [11], pre-load volume, myocardial contractile reserve, circadian hormonal secretion [12] are potential pathways that could participate to BPV. Also, extrinsic factors such as daily activities, stress and smoking must probably participate to BPV in patients with systolic CHF.

### Paradoxical prognostic of BPV: one more Paradigm in CHF

For the last three decades, several studies demonstrated that usual cardiovascular risk factors become good prognosis markers in patients with CHF. Thus, high body mass index [13], hypercholesterolemia or high BP are associated with survival in a CHF population [14]. If BPV is added to lots of complex physiological adaptive system to survive [10], a high level of it worse the prognostic of hypertensive patients [15] whereas a very low level worse the prognosis of CHF. That is one more paradigm in CHF and BPV must be took into account among the reverse epidemiology of conventional risk factors in CHF [16].

## Conclusion

BPV is simple tool, easily available, predictor of cardiac death and/or cardiac transplantation in patients with systolic HF. Measure of BPV could help physicians to improve the management of patients with HF by helping to better assess the prognosis and adjust the therapeutics.

## Abbreviations

ABPM, ambulatory blood pressure monitoring; BP, blood pressure; BPV, blood pressure variability; HF, heart failure; PP, pulse pressure
